# EMDS-5: Environmental Microorganism image dataset Fifth Version for multiple image analysis tasks

**DOI:** 10.1371/journal.pone.0250631

**Published:** 2021-05-12

**Authors:** Zihan Li, Chen Li, Yudong Yao, Jinghua Zhang, Md Mamunur Rahaman, Hao Xu, Frank Kulwa, Bolin Lu, Xuemin Zhu, Tao Jiang

**Affiliations:** 1 Microscopic Image and Medical Image Analysis Group, College of Medicine and Biological Information Engineering, Northeastern University, Shenyang, PR China; 2 Department of Electrical and Computer Engineering, Stevens Institute of Technology, Hoboken, NJ, United States of America; 3 School of Biomedical Engineering, Huazhong University of Science and Technology, Wuhan, China; 4 Whiting School of Engineering, Johns Hopkins University, Baltimore, MD, United States of America; 5 School of Control Engineering, Chengdu University of Information Technology, Chengdu, China; Polytechnical Universidad de Madrid, SPAIN

## Abstract

*Environmental Microorganism Data Set Fifth Version* (EMDS-5) is a microscopic image dataset including original *Environmental Microorganism* (EM) images and two sets of *Ground Truth* (GT) images. The GT image sets include a single-object GT image set and a multi-object GT image set. EMDS-5 has 21 types of EMs, each of which contains 20 original EM images, 20 single-object GT images and 20 multi-object GT images. EMDS-5 can realize to evaluate image preprocessing, image segmentation, feature extraction, image classification and image retrieval functions. In order to prove the effectiveness of EMDS-5, for each function, we select the most representative algorithms and price indicators for testing and evaluation. The image preprocessing functions contain two parts: image denoising and image edge detection. Image denoising uses nine kinds of filters to denoise 13 kinds of noises, respectively. In the aspect of edge detection, six edge detection operators are used to detect the edges of the images, and two evaluation indicators, peak-signal to noise ratio and mean structural similarity, are used for evaluation. Image segmentation includes single-object image segmentation and multi-object image segmentation. Six methods are used for single-object image segmentation, while *k*-means and U-net are used for multi-object segmentation. We extract nine features from the images in EMDS-5 and use the Support Vector Machine (SVM) classifier for testing. In terms of image classification, we select the VGG16 feature to test SVM, *k*-Nearest Neighbors, Random Forests. We test two types of retrieval approaches: texture feature retrieval and deep learning feature retrieval. We select the last layer of features of VGG16 network and ResNet50 network as feature vectors. We use mean average precision as the evaluation index for retrieval. EMDS-5 is available at the URL:https://github.com/NEUZihan/EMDS-5.git.

## 1 Introduction

### 1.1 Environmental Microorganisms

**Fig 1:**
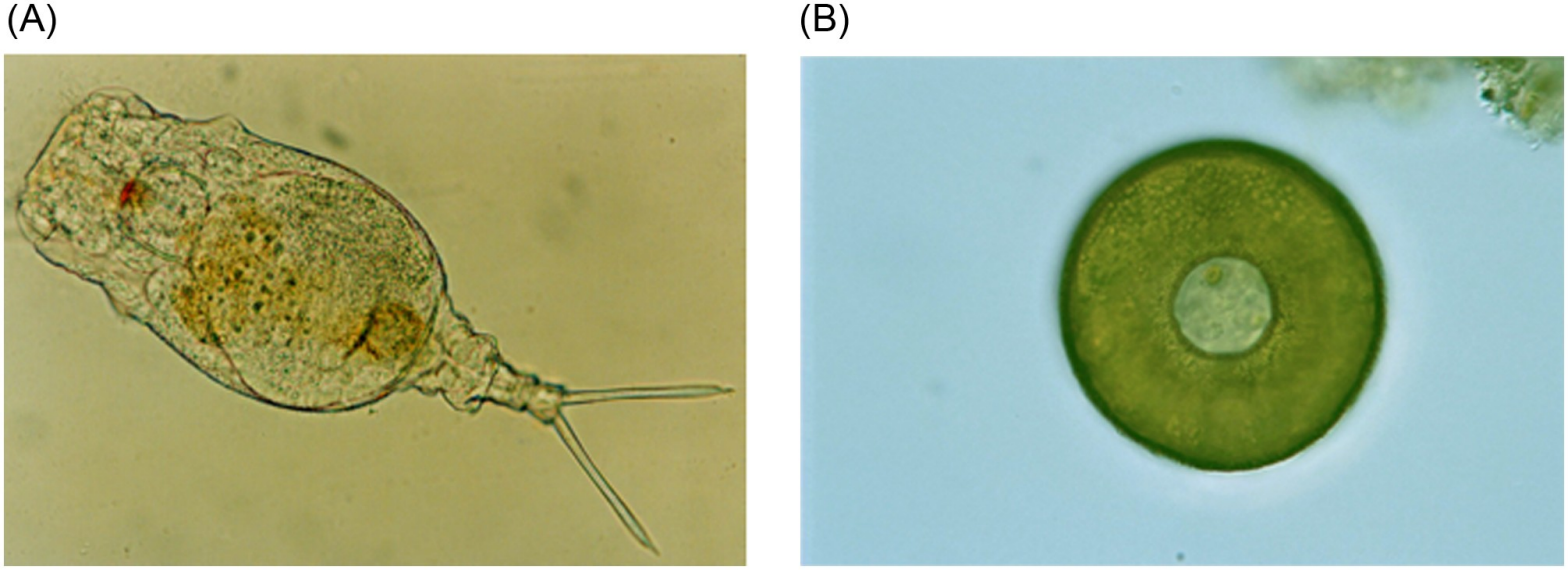
An example of EM images.

All the time, *Environmental Microorganisms* (EMs) [1] are part of our environment. Some EMs bring us benefits, while others affect our physical health. Many researchers devote themselves to study these microorganisms to improve our lives. Nowadays we usually use a microscope to observe EMs. However, scholars sometimes get it wrongly. Image analysis has a great significance for the analysis of EM images. It can help researchers to analyze the types and forms of EMs. For example, *Rotifera* is a common EM and it is widely distributed in lakes, ponds, rivers and other brackish water bodies, having great significance in the study of ecosystem structure function and biological productivity because of their extremely fast reproduction rate and high yield. In addition, *Arcella* is also a kind of common EMs. *Arcella* mainly feeds on plant giardia and single-celled algae. An oligoplastic water body is the most suitable living environment. Two EM image examples are shown in Fig 1.

### 1.2 Application scenarios of Environmental Microorganisms

Noise can be generated during the acquisition or transmission of digital images [2]. Image denoising can reduce the noise of EM images while preserving the details. In addition, image segmentation is the technique and process of dividing an image into a number of specific regions with unique properties and extracting specific targets [3]. So image segmentation technology can be used to segment images of EMs to separate microorganisms from the complex background of the images. After that, the feature extraction part is performed. When the input data is too complex or the amount of data to be processed is large and redundant, the input data will be converted into streamlined features (such as the commonly used ‘feature vectors’). Feature extraction is the process of transforming redundant input data into the desired streamlined features [4]. For the segmented EMs, we usually extract their shape features, color features or deep learning features. We need to use these features for image classification and image retrieval. Image classification is determined by the trained classifier, which is trained by the training data with category labels. We put the extracted feature vectors into a classifier and match them with the known data and put them into the same group of EMs. Image retrieval is given a query image and searches for similar images. We extract the feature vector and calculate its similarity to the feature vector of the known data.

### 1.3 Contribution

Environmental surveys are always carried out in an outdoor environment where conditions such as temperature and salinity are constantly changing. As EMs are very sensitive to these environmental conditions the quality of the observed EMs can be easily affected. It is difficult to collect sufficient EM images [5]. As a result, when researchers want to create EM datasets, they often run out of data. Currently, there are some existing EM datasets, but many of them are not open source. To the best of our knowledge, we know seven special EM datasets. This will make it difficult for EM researchers to obtain the existing EM data set and require much time to collect it. In two cases, we only know the types of microorganisms and the number of samples used in user experiments. The remaining five are our EMDS series. The seven databases are NMCR [6], CECC [7], EMDS-1 [8–10], EMDS-2 [8–12], EMDS-3 [1, 13, 14], EMDS-4 [15–18] and EMDS-5 [19, 20]. *Environmental Microorganism Data Set Fifth Version* (EMDS-5) has been made available to other researchers as an open source dataset. In addition, EMDS-5 has many advantages over other datasets. EMDS-5 provides the corresponding *Ground Truth* (GT) images. Since it takes a lot of time and human resources to make GT images, many datasets do not make GT images corresponding to their own data sets. GT images play an important role in image analysis. GT images can be a significant evaluation index for image segmentation. The result of image segmentation can be judged by comparing the segmented image with the GT images. EDMS-5 has a variety of EM images to provide sufficient data support for image classification and image retrieval. The experiment of multi-classification can be carried out for image classification of multi-species EMs to obtain ideal results. At the same time, many kinds of EM images and sufficient data provide strong data support for the results of image retrieval.

## 2 Dataset information of EMDS-5

**Fig 2:**
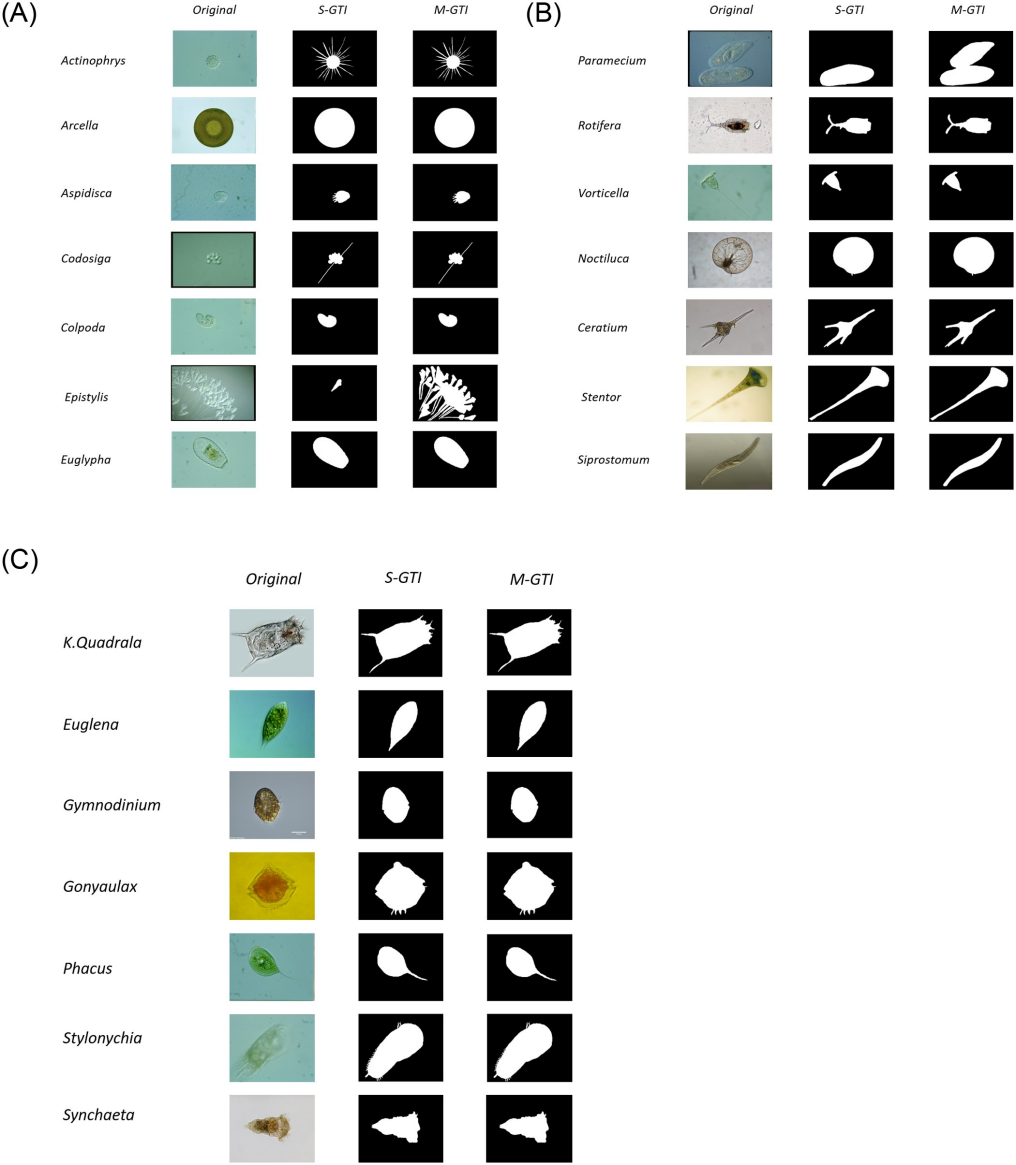
An example of 21 EM classes in EMDS-5. Single-object GT images (SGI), Multi-object GT images (MGI).

EMDS-5 is made up of 1260 images of 21 EM classes. The original 420 EM images are partly collected under artificial light sources and partly under natural light sources with a 400× optical microscope. In addition, 840 GT images are manually prepared, including 420 single-object GT images and 420 multi-object GT images. Basic information of the 21 EM classes in EMDS-5 is given in Table 1, and an example of 21 EM classes in EMDS-5 is shown in Fig 2.

**Table 1:** Basic information of 21 EM classes in EMDS-5. Number of original images (NoOI), Number of single-object GT images (NoSGI), Number of multi-object GT images (NoMGI), Visible characteristics (VC).

Classes	NoOI	NoSGI	NoMGI	VC	Classes	NoOI	NoSGI	NoMGI	VC
*Actinophrys*	20	20	20	Spherical	*Ceratium*	20	20	20	Ring or slightly spiral
*Arcella*	20	20	20	Ellipsoid	*Stentor*	20	20	20	Trumpet
*Aspidisca*	20	20	20	Parasiticon skin lesions of Turbot	*Siprostomum*	20	20	20	Worm-like
*Codosiga*	20	20	20	Each cell has a flagella	*K. Quadrala*	20	20	20	Ellipsoid
*Colpoda*	20	20	20	Kidney	*Euglena*	20	20	20	Phototaxis
*Epistylis*	20	20	20	Funnel shape	*Gymnodinium*	20	20	20	Spherical or oval
*Euglypha*	20	20	20	Oval	*Gonyaulax*	20	20	20	Covered by tightly bonded cellulosic plates
*Paramecium*	20	20	20	Sole shape	*Phacus*	20	20	20	Dorsal ventral flat
*Rotifera*	20	20	20	Roulette composed of cilia	*Stylonychia*	20	20	20	Fan shaped
*Vorticlla*	20	20	20	Dendritic	*Synchaeta*	20	20	20	Transparent and flexible
*Noctiluca*	20	20	20	With luminous ability	-	-	-	-	-
Total	420	420	420	-	Total	420	420	420	-

Three researches from University of Science and Technology Beijing (China) and University of Heidelberg (Germany) provide the original image data of EMDS-5. Furthermore, the preparation of EMDS-5 GT images is jointly completed by three researchers from Northeastern University (China), Johns Hopkins University (US) and Huazhong University of Science and Technology (China). All of them have research backgrounds in Environmental Engineering or Biological Information Engineering. Especially, EMDS-5 GT images are manually labelled based on pixel-level with two rules:

- Rule A: The area where an EM is located is labelled as foreground (1, white). In contrast, other areas are labelled as background (0, black).
- Rule B: Because the microscopic images in EMDS-5 are collected under optical microscopes, this process produces interference fringes and results in unwanted edges in the EM images. Hence, when making GT images, the most complicated thing is to determine the edges of an EM. First, each researcher selects the edges that she or he thinks are the clearest to label. Then, if their labelling results are conflict, they have a collective discussion to judge and decide a final solution.

## 3 Image processing evaluation using EMDS-5

### 3.1 Evaluation of image denoising methods

**Fig 3:**
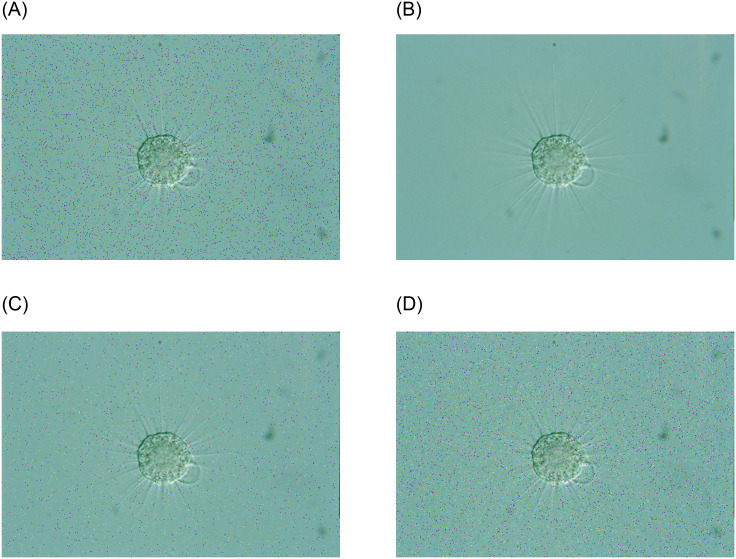
An example of different noisy EM images using EMDS-5 images.

We add a total of 13 kinds of noise to the original images and then denoise the noisy images with different methods. The noise we have chosen is grouped into Poisson noise, multiplicative noise, Gaussian noise, and pretzel noise in total. Gaussian noise is a noise whose probability density function follows a normal distribution. Poisson noise is a noise model that conforms to the Poisson distribution. Multiplicative noise is a type of noise caused by random variations in channel characteristics. Multiplicative noise is related to the signal by multiplication. Pepper noise, also known as impulse noise, which randomly changes some pixel values, is a black and white bright and dark dot noise generated by the image sensor, transmission channel, decoding process, etc. For multiplicative noise, we divide it into multiplicative noise with a variance of 0.2 and multiplicative noise with a variance of 0.04 according to the variance of multiplicative noise. We classify Gaussian noise according to the mean and variance: Gaussian noise with mean 0 variance 0.01, mean 0.5 variance 0.01, mean 0 variance 0.03 and mean 0.5 variance 0.03, and also Position Gaussian noise and Brightness Gaussian noise. Similarly, we divide the pepper noise into pepper noise, salt noise, pepper noise with a density of 0.01 and pepper noise with a density of 0.03. We summarize the above noises and count them into 13 kinds of noise to add noise to the original image respectively, and then use different filters for denoising. An example of the noisy EM images is shown in Fig 3.

We use nine different methods to denoise and choose to use the similarity between the denoised image and the original image and the variance of the two as the evaluation index. The evaluation index is expressed by Eq (1) [2].

$$\begin{array}{c} A=1-\frac{\sum_{1}^{n}|i_{1}-i|}{N\times 255}, \end{array}$$ (1)

where *A* is the similarity, *i*_1_ is the denoised image, *i* is the original image, and *N* is the number of pixels. The closer the value of *A* is to 1, the better the denoising effect. We use the above original image as an example, and use the table to list the similarity between the image after removing various noises and the original image using different filters. Here we have simplified the names of the noise and filters involved in the table as follows.*Types of noise* (ToN), *Denoising method* (DM), *Two-Dimensional Rank Order Filter* (TROF), *Mean Filter Window*: 3 × 3 (MF: 3 × 3), *Mean Filter Window*: 5 × 5 (MF: 5 × 5), *Wiener Filter Window*: 3 × 3 (WF: 3 × 3), *Wiener Filter Window*: 5 × 5 (WF: 5 × 5), *Maximum Filter* (MaxF), *Minimum Filter* (MinF), *Geometric Mean Filter* (GMF), *Arithmetic Mean Filter* (AMF), *Poisson noise* (PN), *Multiplicative noise variance: 0.2* (MN *v*: 0.2), *Multiplicative noise variance: 0.04* (MN *v*: 0.04), *Gaussian noise Variance: 0.01, Mean: 0* (GN *m*: 0, *v*: 0.01), *Gaussian noise Variance: 0.01, Mean: 0.5* (GN m: 0.5, *v*: 0.01), *Gaussian noise Variance: 0.03, Mean: 0* (GN *m*: 0, *v*: 0.03), *Gaussian noise Variance: 0.03, Mean: 0.5* (GN m: 0.5, *v*: 0.03), *Salt and pepper noise density: 0.01* (SPN *d*: 0.01), *Salt and pepper noise density: 0.03* (SPN *d*: 0.03), *Pepper noise* (PpN), *Brightness Gaussian noise* (BGN), *Position Gaussian noise* (PGN), *Salt noise* (SN). The comparison of similarities between denoised images and original image using EMDS-5 are shown in Table 2.

**Table 2:** A comparison of similarities between denoised images and original image using EMDS-5. (In [%].).

ToN / DM	TROF	MF: 3 × 3	MF: 5 × 5	WF: 3 × 3	WF: 5 × 5	MaxF	MinF	GMF	AMF
PN	99.47	99.17	99.39	99.26	99.47	92.56	99.98	99.65	99.30
MN, *v*: 0.2	96.57	94.34	96.43	96.56	98.11	68.55	99.90	98.61	97.08
MN, *v*: 0.04	98.27	97.14	98.16	97.91	98.73	81.13	99.94	98.66	98.21
GN, *m*: 0, *v*: 0.01	98.99	98.34	98.94	98.41	98.99	84.99	99.97	98.88	98.64
GN, *m*: 0.5, *v*: 0.01	61.51	61.13	60.96	62.05	62.05	60.18	64.50	62.10	62.35
GN, *m*: 0, *v*: 0.03	98.33	97.21	98.21	97.32	98.36	75.24	99.95	98.54	97.81
GN, *m*: 0.5, *v*: 0.03	62.00	61.56	61.21	63.95	63.96	60.18	68.78	64.22	64.23
SPN, *d*: 0.01	99.77	99.79	99.69	99.60	99.57	97.00	99.98	99.53	99.59
SPN, *d*: 0.03	99.77	99.79	99.69	99.25	99.29	93.86	99.98	99.31	99.30
PpN	99.78	99.80	99.70	99.81	99.80	98.77	99.98	99.70	99.80
BGN	99.32	98.94	99.26	99.03	99.36	90.54	99.98	98.93	99.13
PGN	99.05	98.44	98.98	98.33	98.81	85.86	99.97	99.16	98.70
SN	99.79	99.81	99.71	99.82	99.84	98.77	99.98	98.77	99.82

From the comparision in Table 2, we find that EMDS-5 can support distinguishable evaluation for different denoising methods. For example, the maximum filtering effect is not very good, so it is not ideal for the denoising results of Gaussian noise and multiplicative noise, but it is still very good for the denoising results of salt and pepper noise and Poisson noise. In addition, the mean variance of the denoised image and the original image is an indicator of stability of denoising mehods. The mean variance is expressed by Eq (2) [2].

$$\begin{array}{c} S=\frac{\sum_{1}^{n}(l_{\left(i,j\right)}-B_{\left(i,j\right)}{)}^{2}}{\sum_{1}^{n}l_{\left(i,j\right)}^{2}}, \end{array}$$ (2)

where *l*_(*i*, *j*)_ and *B*_(*i*, *j*)_ represent the pixels corresponding to the original image after denoising, and *S* represents the mean variance. The comparison of variances between denoised images and original image using EMDS-5 are shown in Table 3.

**Table 3:** A comparison of variances between denoised images and original image using EMDS-5. (In [%].).

ToN / DM	TROF	MF: 3 × 3	MF: 5 × 5	WF: 3 × 3	WF: 5 × 5	MaxF	MinF	GMF	AMF
PN	0.57	0.13	0.10	0.10	0.06	1.81	1.71	0.07	0.19
MN, *v*: 0.2	5.13	5.54	2.53	2.68	1.14	27.70	25.82	3.69	2.17
MN, *v*: 0.04	1.64	1.41	0.67	0.75	0.34	10.39	7.21	0.68	0.67
GN, *m*: 0, *v*: 0.01	0.85	0.49	0.25	0.48	0.22	7.10	3.94	0.40	0.42
GN, *m*: 0.5, *v*: 0.01	42.16	42.41	42.85	40.15	40.13	44.96	35.96	40.02	39.78
GN, *m*: 0, *v*: 0.03	1.60	1.41	0.64	1.39	0.60	18.72	9.81	1.87	0.99
GN, *m*: 0.5, *v*: 0.03	41.04	41.47	42.29	36.38	36.15	44.97	28.71	35.67	35.92
SPN, *d*: 0.01	0.49	0.02	0.05	0.59	0.39	2.20	1.11	4.53	0.20
SPN, *d*: 0.03	0.48	0.02	0.05	1.35	0.69	5.87	1.80	12.67	0.37
PpN	0.63	0.03	0.05	1.42	0.71	0.16	2.39	16.78	0.39
BGN	0.63	0.21	0.13	0.18	0.10	2.89	2.19	0.43	0.24
PGN	0.86	0.49	0.25	0.67	0.39	7.02	3.96	0.20	0.42
SN	0.49	0.03	0.06	1.84	0.84	0.16	3.74	0.18	0.54

From the comparison in Table 3, we find that our EMDS-5 is useful to test and evaluate image decisioning methods effectively. For example, increasing the mean value of Gaussian noise will result in greater variance between the denoised images and the original images, indicating that the results after denoising are not very stable.

In addition, we chose to use the most well-known *Image Quality Assessment* (IQA) to evaluate the image quality of the denoised images. Here we use two indicators, *Peak-Signal to Noise Ratio* (PSNR) and *Mean Structural Similarity* (SSIM), to evaluate the image quality of the denoised images. PSNR calculates the difference between the grey value of the pixel to be measured and the corresponding pixel of the reference image. PSNR is a method of assessing image quality using a statistical approach. We hypothesis that the image to be evaluated is *F*, the reference image is *R*, and their sizes are *MN*. The calculation method for characterizing image quality using PSNR, which is expressed by Eq (3) [21].

$$\begin{array}{c} \text{PSNR}=10\;\text{lg}\frac{255^{2}}{\frac{1}{\text{MN}}\sum_{i=1}^{M}\sum_{j=1}^{N}{\left|R\left(i,j\right)-F\left(i,j\right)\right|}^{2}}. \end{array}$$ (3)

PSNR measures the image quality by calculating the global size of the pixel error between the image to be evaluated and the reference image. The larger the PSNR value, the less distortion between the image to be evaluated and the reference image, and the image quality is better. SSIM is a commonly used image quality evaluation method originally proposed in [22]. SSIM is composed of three contrast functions. The brightness contrast function is expressed by Eq (4).

$$\begin{array}{c} l\left(x,y\right)=\frac{2u_{x}u_{y}+c_{1}}{u_{x}^{2}+u_{y}^{2}+c_{1}}. \end{array}$$ (4)

Contrast contrast function is expressed by Eq (5).

$$\begin{array}{c} c\left(x,y\right)=\frac{2\sigma_{x}\sigma_{y}+c_{2}}{\sigma_{x}^{2}+\sigma_{y}^{2}+c_{2}}. \end{array}$$ (5)

Structural contrast function is expressed by Eq (6).

$$\begin{array}{c} s\left(x,y\right)=\frac{\sigma_{xy}+c_{3}}{\sigma_{x}\sigma_{y}+c_{3}}. \end{array}$$ (6)

*σ*_*xy*_ is expressed by Eq (7).

$$\begin{array}{c} \sigma_{xy}=\frac{1}{N-1}\sum_{i=1}^{N}(x_{i}-\mu_{x})(y_{i}-\mu_{y}). \end{array}$$ (7)

We combine the three functions and finally get the SSIM index function expressed by Eq (8).

$$\begin{array}{c} \mathrm{SSIM}\left(x,y\right)=l{\left(x,y\right)}^{a}\cdot c{\left(x,y\right)}^{\beta }\cdot s{\left(x,y\right)}^{\Gamma }. \end{array}$$ (8)

Where *u*_*x*_, *u*_*y*_ are all pixels of the image block; *σ*_*x*_, *σ*_*y*_ are the standard deviation of the image pixel values; *σ*_*x*_ *σ*_*y*_ is the covariance of *x* and *y*; *C*_1_, *C*_2_, *C*_3_ are constants, in order to avoid the system error caused when the denominator is 0. SSIM is a number between 0 and 1. The larger the SSIM, the smaller the difference between the two images. The comparison of PSNR between denoised images and original image using EMDS-5 are shown in Table 4.

**Table 4:** A comparison of PSNR between denoised images and original image using EMDS-5.

ToN / DM	TROF	MF: 3 × 3	MF: 5 × 5	WF: 3 × 3	WF: 5 × 5	MaxF	MinF	GMF	AMF
PN	26.64	31.86	31.79	33.42	34.20	20.89	21.90	33.12	30.65
MN, *v*: 0.2	17.76	17.69	20.63	19.77	22.39	11.07	10.76	18.78	21.05
MN, *v*: 0.04	16.16	27.77	25.42	25.65	23.25	25.61	22.38	15.01	25.98
GN, *m*: 0, *v*: 0.01	24.69	26.82	28.55	27.21	29.48	15.67	18.20	26.23	27.31
GN, *m*: 0.5, *v*: 0.01	8.25	37.75	8.21	8.44	8.44	7.46	9.15	8.47	8.47
GN, *m*: 0, *v*: 0.03	22.01	22.64	23.90	22.73	25.53	11.86	14.50	20.12	23.90
GN, *m*: 0.5, *v*: 0.03	8.35	8.29	8.23	8.76	8.78	7.25	10.18	8.91	8.81
SPN, *d*: 0.01	27.35	37.62	33.89	26.01	27.59	19.23	23.83	18.26	30.10
SPN, *d*: 0.03	27.33	37.13	33.79	22.38	25.03	15.42	21.81	13.81	27.62
PpN	27.32	37.00	33.73	23.30	25.91	27.06	20.69	12.68	28.14
BGN	25.94	29.70	30.36	30.54	31.96	18.66	20.41	25.96	39.19
PGN	24.72	26.83	28.59	26.07	27.83	15.75	18.20	28.44	27.31
SN	27.30	36.37	33.47	22.16	25.04	27.07	18.73	30.18	26.87

The comparison of SSIM between denoised images and original image using EMDS-5 are shown in Table 5.

**Table 5:** A comparison of SSIM between denoised images and original image using EMDS-5. (In [%].).

ToN / DM	TROF	MF: 3 × 3	MF: 5 × 5	WF: 3 × 3	WF: 5 × 5	MaxF	MinF	GMF	AMF
PN	78.63	78.46	84.67	82.97	89.01	64.35	70.57	94.56	82.53
MN, *v*: 0.2	19.58	18.35	29.28	25.12	40.59	44.06	15.90	26.07	28.91
MN, *v*: 0.04	39.23	66.87	47.44	52.31	36.00	50.92	38.57	54.48	51.62
GN, *m*: 0, *v*: 0.01	55.54	51.64	69.55	54.52	74.20	37.11	42.61	56.75	59.47
GN, *m*: 0.5, *v*: 0.01	61.87	95.79	65.46	63.21	67.68	63.21	54.86	64.30	63.92
GN, *m*: 0, *v*: 0.03	34.54	30.69	50.24	32.64	53.63	25.99	23.14	30.66	38.48
GN, *m*: 0.5, *v*: 0.03	54.98	55.18	61.14	51.10	61.90	63.17	36.86	53.73	54.66
SPN, *d*: 0.01	91.54	95.75	91.21	72.62	75.85	61.28	89.06	59.04	83.39
SPN, *d*: 0.03	91.47	95.64	91.17	49.01	61.71	33.00	79.00	27.90	67.20
PpN	91.49	95.67	91.18	54.58	64.92	90.00	72.14	21.98	72.10
BGN	71.18	69.21	79.99	72.88	84.17	52.18	60.77	59.74	74.71
PGN	58.65	55.57	71.01	56.44	69.63	41.91	46.91	73.03	62.46
SN	91.45	95.56	91.12	46.09	62.75	90.55	52.56	81.52	65.01

### 3.2 Evaluation of edge detection methods

**Fig 4:**
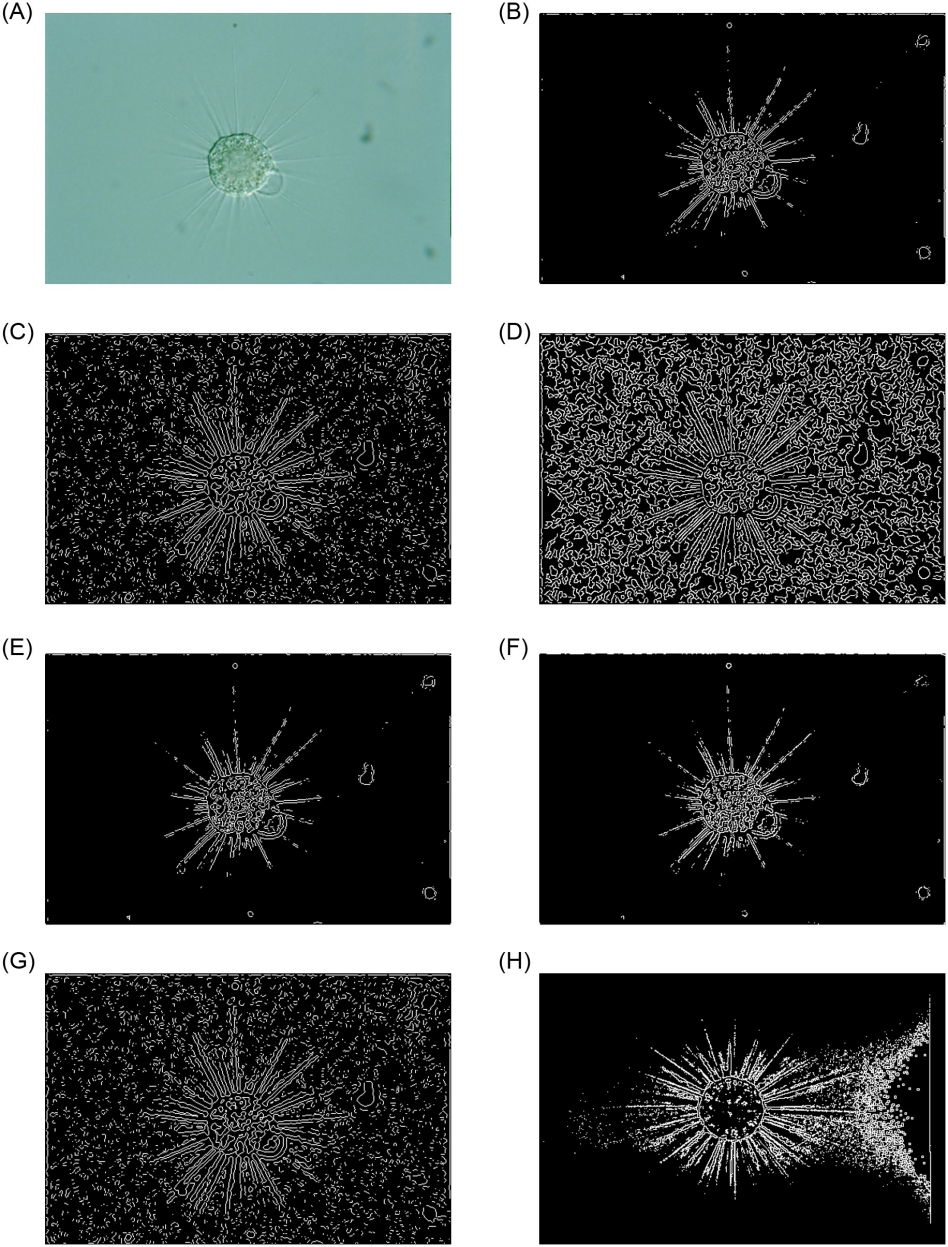
An example of seven edge detection results using EMDS-5 images.

Edge detection is an important component of image preprocessing. In order to prove the effectiveness of our EMDS-5 in edge detecition evaluation, seven operators are used to detect edges from images in EMDS-5 dataset. The seven operators are Canny, Laplace of Gaussian (LoG), Prewitt, Roberts, Sobel, Zero cross and CNN, and an example of the edge detection results is shown in Fig 4.

For the edge detection of images, we choose two evaluation metrics, PSNR and SSIM, to evaluate the results of edge detection. We choose the edge detection result obtained by Sobel operator as the standard, and compare the results obtained by other edge detection methods with it and evaluate the results.

A comparison of edge detection methods using EMDS-5 is shown in Table 6.

**Table 6:** A comparison of edge detection methods using EMDS-5. Evaluation index (EI), Operator type (OT).

EI / OT	Canny	LoG	Prewitt	Roberts	Zero cross	CNN
*PSNR*	54.84	58.16	72.44	63.37	58.16	13.97
*SSIM*	98.89%	99.67%	99.99%	99.94%	99.67%	76.11%

From Table 6, we find that the PSNR evaluation index that the edge detection results obtained by the Prewitt operator are the most similar to the Sobel results. The SSIM evaluation index shows that the difference between the results of other operators and the results of Sobel operator is also very small. By comparison, we can see that EMDS-5 images can be used to detect and evaluate various edge detection methods.

## 4 Image segmentation evaluation using EMDS-5

### 4.1 Single-object image segmentation

**Fig 5:**
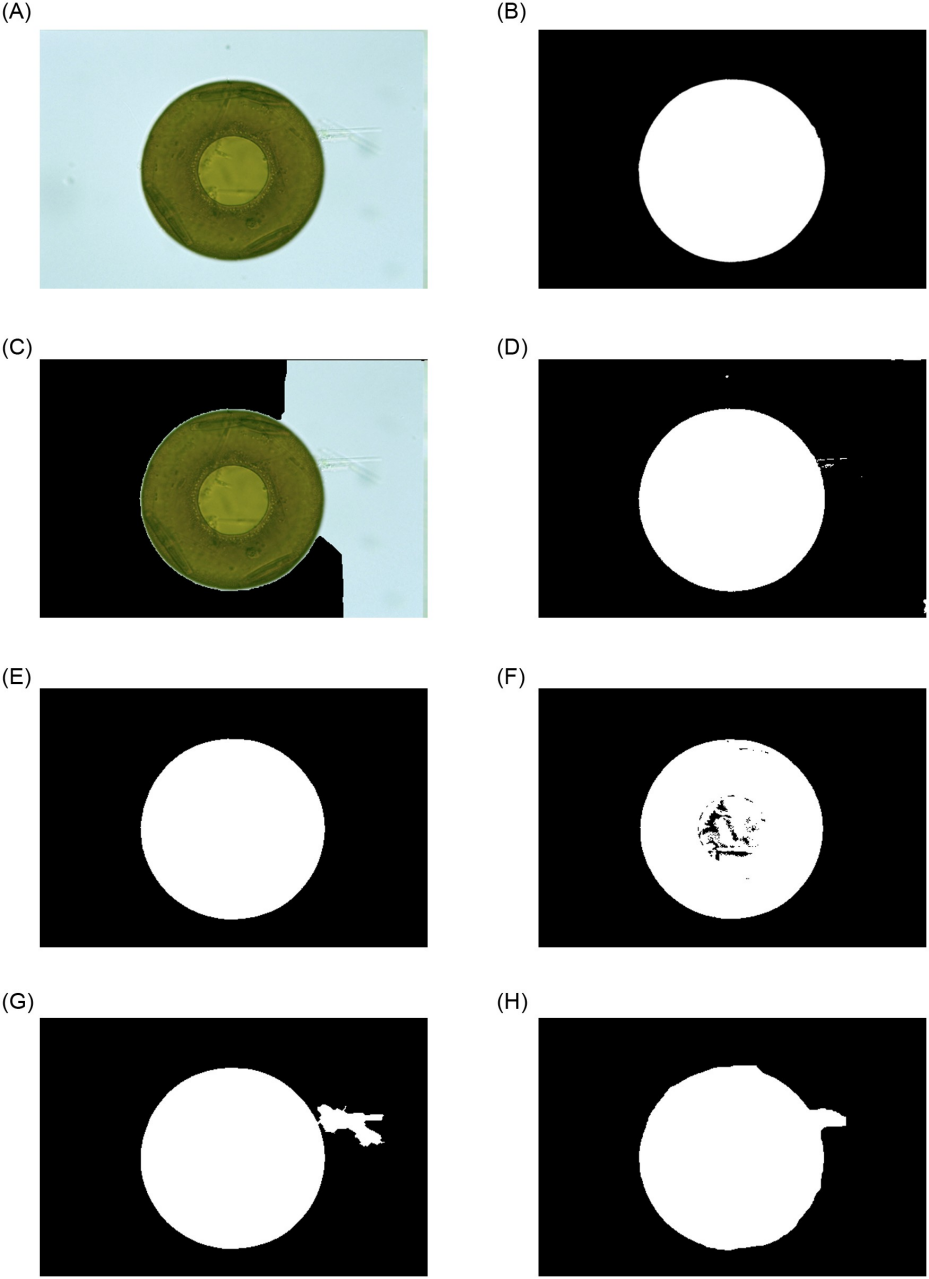
An example of different single-object segmentation results using EMDS-5 images.

In order to prove the effectiveness of EMDS-5 for image segmentation evaluation, six typical image segmentation methods are compared to segment the EMDS-5 original images, including GrubCut, Markov Random Field (MRF), Canny edge detection based, Watershed, Otsu thresholding and Region growing approaches. GrubCut is a common and classic method of semi-automatic segmentation. MRF is a classical graph based segmentation method. Otsu thresholding is an image segmentation method based on threshold. Region growing approaches and Watershed algorithm are classical region based segmentation methods. An example of different single-object segmentation results is shown in Fig 5.

We compare the images obtained after image segmentation with the corresponding GT images, where five evaluation indexes in Table 7 are used to evaluate the segmentation results [23, 24].

**Table 7:** The image segmentation evaluation metrics used in this paper and their definitions. TP (True Positive), FN (False Negative), FP (False Positive).

Metric	Definition
Dice	$\text{Dice}=\frac{2\times |V_{\text{pred}\;}\cap V_{\text{gt}}|}{\left|V_{\text{pred}\;}|+|V_{\text{gt}}\right|}$
Jaccard	$\text{Jaccard}=\frac{\left|V_{\text{pred}}\cap V_{\text{gt}}\right|}{\left|V_{\text{pred}}\cup V_{\text{gt}}\right|}$
Recall	$\text{Recall}=\frac{\text{TP}}{\text{TP}+\text{FN}}$

In Table 7, *V*_pred_ represents the foreground that is predicted by the model; *V*_gt_ represents the foreground in a ground truth image. We show the evaluation results of the sample images in Table 8.

**Table 8:** A comparison of single-object segmentation methods using EMDS-5. Image segmentation methods (ISM), Evaluation index (EI), Watershed algorithm (WA), Otsu thresholding (OT), Region growing (RG). (In [%].).

ISM / EI	Dice	Jaccard	Recall
GrubCut	18.41	10.14	10.18
MRF	98.01	96.09	99.67
Canny	59.59	51.48	94.99
WA	57.79	49.50	76.26
OT	98.87	97.76	98.16
RG	86.67	76.47	77.65

From Table 8, it is observed that because the GrubCut method segments the original images, when compared with the GT image it leads to a low evaluation result. Among several other classic single-object image segmentation methods, the results of Otsu Thresholding and MRF segmentation closest to the GT images and the best effect. Other segmentation methods have a certain gap compared with these two segmentation methods. Through the comparison of these image segmentation parties, we can conclude that EMDS-5 is effective in testing and evaluating image segmentation methods.

### 4.2 Multi-object image segmentation

**Fig 6:**
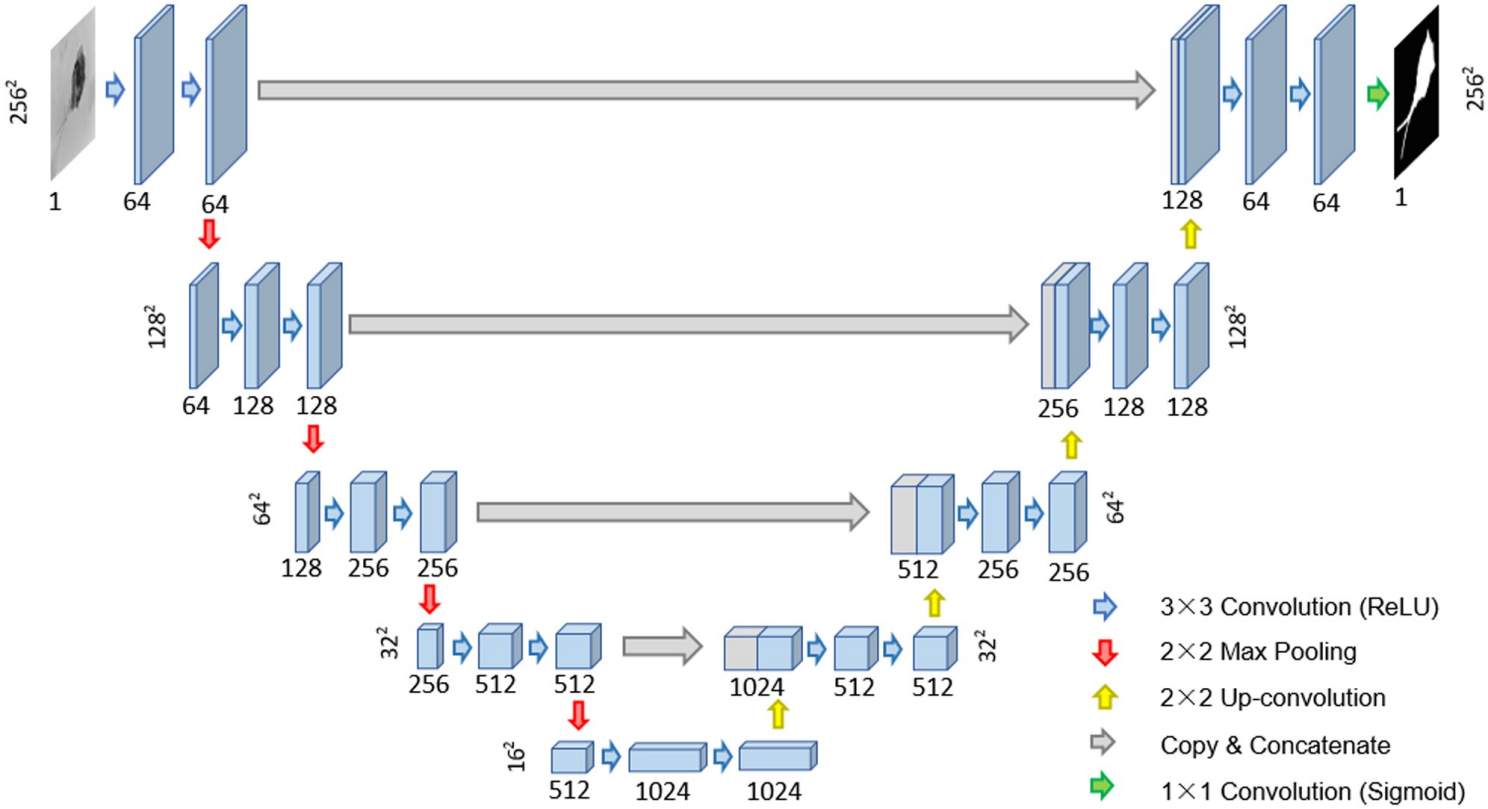
The structure of U-Net.

For multi-object image segmentation, we use two methods, *k*-means and U-net, to test our EMDS-5. *k*-means is an unsupervised learning approach (clustering) and U-net is a supervised learning method (deep convolutional neural network, DCNN). These two methods are representative of the classic methods in their respective fields. The structure of U-net is shown in Fig 6 [19].

U-Net is initially a DCNN for performing microscopic image segmentation tasks. A strong use of data enhancement is at the core of U-net. This allows for more efficient use of existing annotation samples. In addition, U-Net’s end-to-end architecture allows retrieval of shallow information about the network. The structure of U-Net is symmetrical. The U-Net uses a network structure that contains both downsampling and upsampling [24]. The left half is the contracting path, a downsampling operation in which two 3 × 3 convolutions (unfilled convolutions) are repeated, followed by a ReLU activation function with a 2 × 2 maximum pooling layer for downsampling, doubling the number of feature channels at each downsampling, to achieve a minimum resolution of 32 × 32. The right half of the region is the expansive path. There are still a large number of feature channels in the upsampling part, which allow the network to propagate contextual information to the high resolution layers, so that the expansive path is more or less symmetrical with respect to the systolic path, resulting in an U-shaped structure. Each layer in this region contains a 2 × 2 inverse convolution operation for upsampling, which halves the feature channels. A fusion operation with the clipped feature map of the same dimensional layer is also included, followed by the addition of two 3 × 3 convolutions with ReLU activation functions [24]. Since unfilled convolution is used, boundary pixels are lost in each convolution, so cropping is necessary. In the last layer, each 64-component feature vector is mapped to the desired number of classes using 1 × 1 convolution [25].

**Fig 7:**
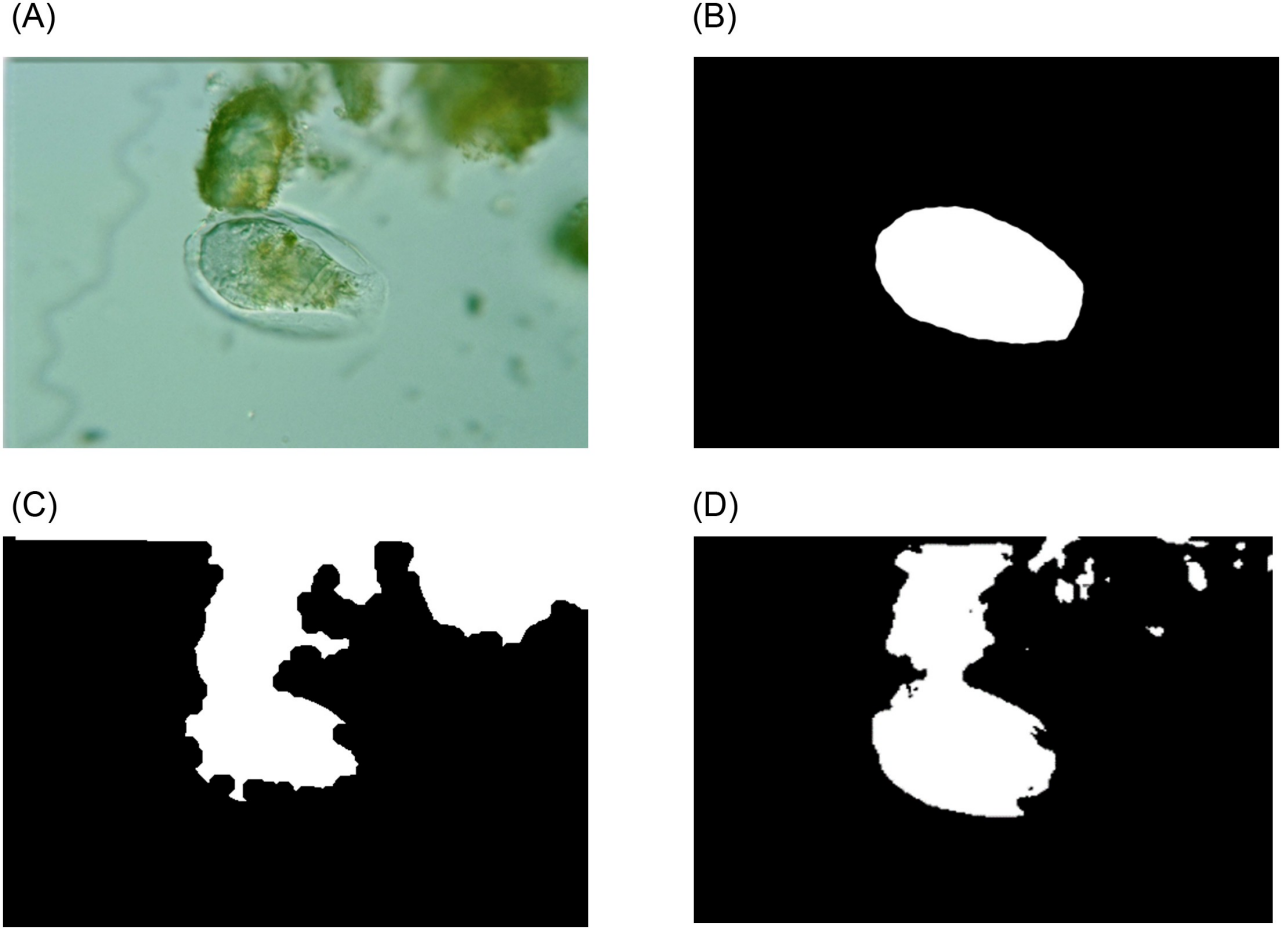
An example of different multi-object segmentation results using EMDS-5.

The examples of different multi-object segmentation results are shown in Fig 7.

For these two multi-object image segmentation methods, a comparison is shown in Table 9.

**Table 9:** A comparison of multi-object segmentation methods using EMDS-5. Image segmentation methods (ISM), Evaluation index (EI). (In [%].).

ISM / EI	Dice	Jaccard	Recall
*k*-means	31.97	25.93	65.81
U-net	85.24	77.41	82.28

It can be seen from Table 9 that the segmentation effect of U-Net in the multi-target image segmentation method is much higher than that of *k*-means, showing the effectiveness of EMDS-5 for evaluaiton of multi-object image segmentation methods.

## 5 Feature extraction evaluation using EMDS-5

**Fig 8:**
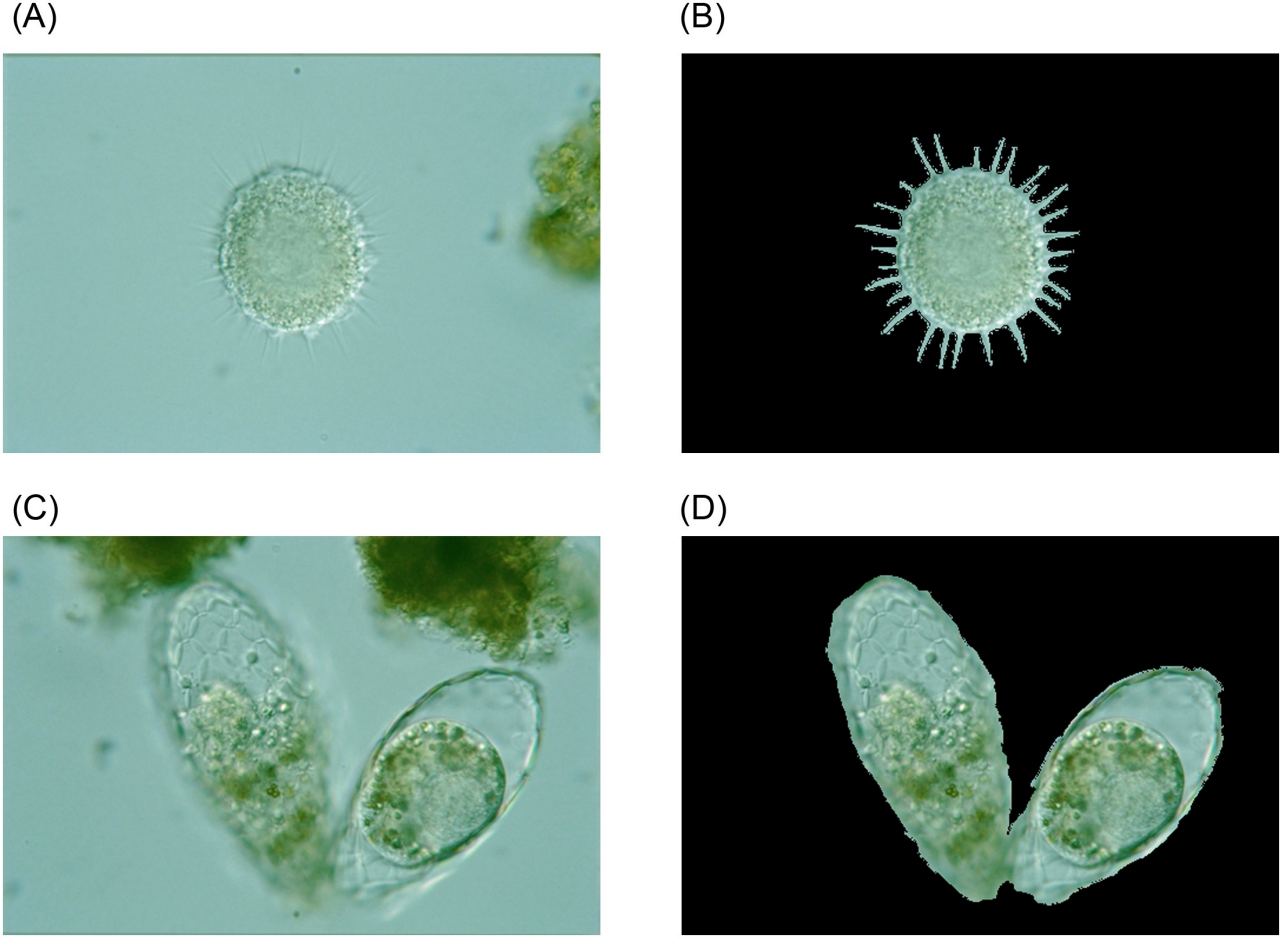
An example of localized EMs by GT images.

We use GT images to localize the target EMs in the original images to test feature extraction methods. Since GT images have single-object GT images and multi-object GT images, feature extraction methods are grouped into two types. An example of original images and target EM images extracted from GT images are shown in Fig 8. First, we randomly select ten images from each EM class as the training set and the other ten as the test set. Then, we extract and compare 12 features, including two color feaures (HSV (Hue, Saturation and Value) and RGB (Red, Green and Blue) features), three texture features (GLCM (Grey-level Co-occurrence Matrix), HOG (Histogram of Oriented Gridients) and LBP (Local Binary Pattern) features), four geometric features (area, perimeter, long and short axis features), seven invariant moment features (Hu moments), and two deep learning features (VGG16 and Resnet50 features). We test the color features extract from the respective channels of RGB features and HSV features as a single feature vector. Lastly, we use a Radial Basis Function Support Vector Machine (RBFSVM) classifier (supported by LIBSVM [26]) to test each feature and calculate their accuracies. The LIBSVM parameters are set as −*s* 0 −*t* 0 −*c* 2 −*g* 1 −*b* 1.

### 5.1 Single-object feature extraction

In Table 10, the accuracies of EM image classification using single-object features are compared.

**Table 10:** Classification accuracy of single-object features by RBFSVM using EMDS-5. Feature type (FT), Accuracy (Acc), Geometric features (Geo), Hu moments (Hu). (In [%].).

FT	RGB-R	RGB-G	RGB-B	HSV-H	HSV-S	HSV-V
*Acc*	27.62	36.67	34.76	30.48	34.29	39.52
Geo	Hu	LBP	HOG	GLCM	VGG16	Resnet50
41.43	7.62	38.01	10	28.10	83.81	39.45

### 5.2 Multi-object feature extraction

In Table 11, the accuracies of EM image classification using multi-object features are compared.

**Table 11:** Classification accuracy of multi-object features by RBFSVM using EMDS-5. F Feature type (FT), Accuracy (Acc), Geometric features (Geo), Hu moments (Hu). (In [%].).

FT	RGB-R	RGB-G	RGB-B	HSV-H	HSV-S	HSV-V
*Acc*	22.86	29.05	28.57	28.57	29.05	33.81
Geo	Hu	LBP	HOG	GLCM	VGG16	Resnet50
38.10	7.62	37.62	14.76	22.38	68.57	23.33

From Tables 10 and 11, we can find that when using the same RBFSVM classifiers to classify EM images with different features, we obtain different classification results, showing the effectiveness of EMDS-5 for the feature extraction evaluation. Especially, because VGG16 feature achieves the best effect, we chose it in the following section about ‘classification evaluation’.

## 6 Image classification evaluation using EMDS-5

We use the features extracted from the EMDS-5 data to test classification performance of different classifiers. As mentioned in Sec. 5, we use the extracted VGG16 features for testing in this section. The VGG16 feature vector selects the 16th layer feature vector. The dimension is 1 × 1000. First, we randomly select ten images from each EM class as the training set and use another ten as test set. Then, we select 14 normally used classifiers for EM image classification, including four SVMs, three *k*-Nearest Neighbors (KNNs), three Random Forests (RFs), two VGG16 and two Inception-V3 classifiers. We combine and compare the extracted VGG16 features with four classic classifiers. In addition, four deep learning classifiers are directly compared. In VGG16 and Inception-V3, we divide the data into test, validation and test sets. Then we test the accuracy of any two types of EM image classification. We change the ratio of the images owned by these three datasets and test the accuracy, separately. Especially, the parameters of four SVM classifiers are shown in Table 12.

**Table 12:** The parameters of four SVM classifiers for EMDS-5 image classification (supported by LIBSVM).

SVM type	Parameter
SVM: linear	−*s* 0 −*t* 0 −*c* 2 −*g* 1 −*b* 1
SVM: polynomial	−*s* 0 −*t* 1 −*r* 0 −*g* 0.42 −*d* 3
SVM: RBF	−*s* 0 −*t* 2 −*c* 2 −*g* 1 −*b* 1
SVM: sigmoid	−*s* 0 −*t* 3 −*r* 0 −*g* 0.042

Furthermore, a comparison of different classifiers for EM image classification using EMDS-5 is shown in Table 13.

**Table 13:** A comparison of EM image classification results using EMDS-5. Accuracy (Acc), *n*Tree (*n*T), VGG16 (Train: Validation: Test = 1: 1: 2) is VGG16: 1: 1: 2, VGG16 (Train: Validation: Test = 1: 2: 1) is VGG16: 1: 2: 1, Inception-V3 (Train: Validation: Test = 1: 1: 2) is I-V3: 1: 1: 2, Inception-V3 (Train: Validation: Test = 1: 2: 1) is I-V3: 1: 2: 1. (In [%].).

Classifier type	SVM: linear	SVM: polynomial	SVM: RBF	SVM: sigmoid
Acc	68.57	63.81	21.91	5.24
*k*-NN, *k*: 1	*k*-NN, *k*: 5	*k*-NN, *k*: 10	RF, *n*T: 10	RF, *n*T: 20
60.48	52.38	48.10	44.76	47.14
RF, *n*T: 30	VGG16, 1:1:2	VGG16, 1:2:1	I-V3, 1:1:2	I-V3, 1:2:1
55.71	81.61	83.23	89.43	90.49

It can be seen from Table 13 that when using the same feature to test different classifiers. The classification results of the two deep learning networks are the best. From the comparison of the results of different classifiers, we can see that EMDS-5 images can be effectively applied to the testing and evaluation of various classification algorithms.

## 7 Image retrieval evaluation using EMDS-5

We use EMDS-5 for image retrieval. Because we use different features, we group the image retrieval methods into two categories: texture feature and deep learning feature based image retrieval approaches. We use Average Precision (AP) [18] to evaluate the retrieval results. AP is derived from the field of information retrieval and is primarily used to evaluate ranked lists of retrieved samples. The definition of AP in our article is shown in Eq (9).

$$\begin{array}{c} \text{AP}=\frac{\sum_{i=1}^{n}\left(P\left(k\right)\times \text{rel}\left(k\right)\right)}{M}. \end{array}$$ (9)

*M* is the number of related EM images, *P*(*k*) is by considering the cut-off position divided by the *k*th position in the list, and rel(*k*) is an index. The EM image rank in the *k*th position is the target type image, then take 1; otherwise, take 0. AP represents the average value of the accuracy of the current position target type EM image. Our experiment is conducted on 21 types of EM images, so we apply the mean AP (mAP) to summarize the APs of each class. It is calculated by obtaining the average value of APs. During the retrieval process, we match the feature vector of the image to be tested with the feature vectors of all the images in the EMDS-5 dataset and calculate the Euclidean distance between the two. Then calculate the mAP value of the search result of the type of image to be tested as the search result. We display the first 20 images in the search results, in which the frame of the correct image is marked with a color.

### 7.1 Texture feature based image retrieval using EMDS-5

**Fig 9:**
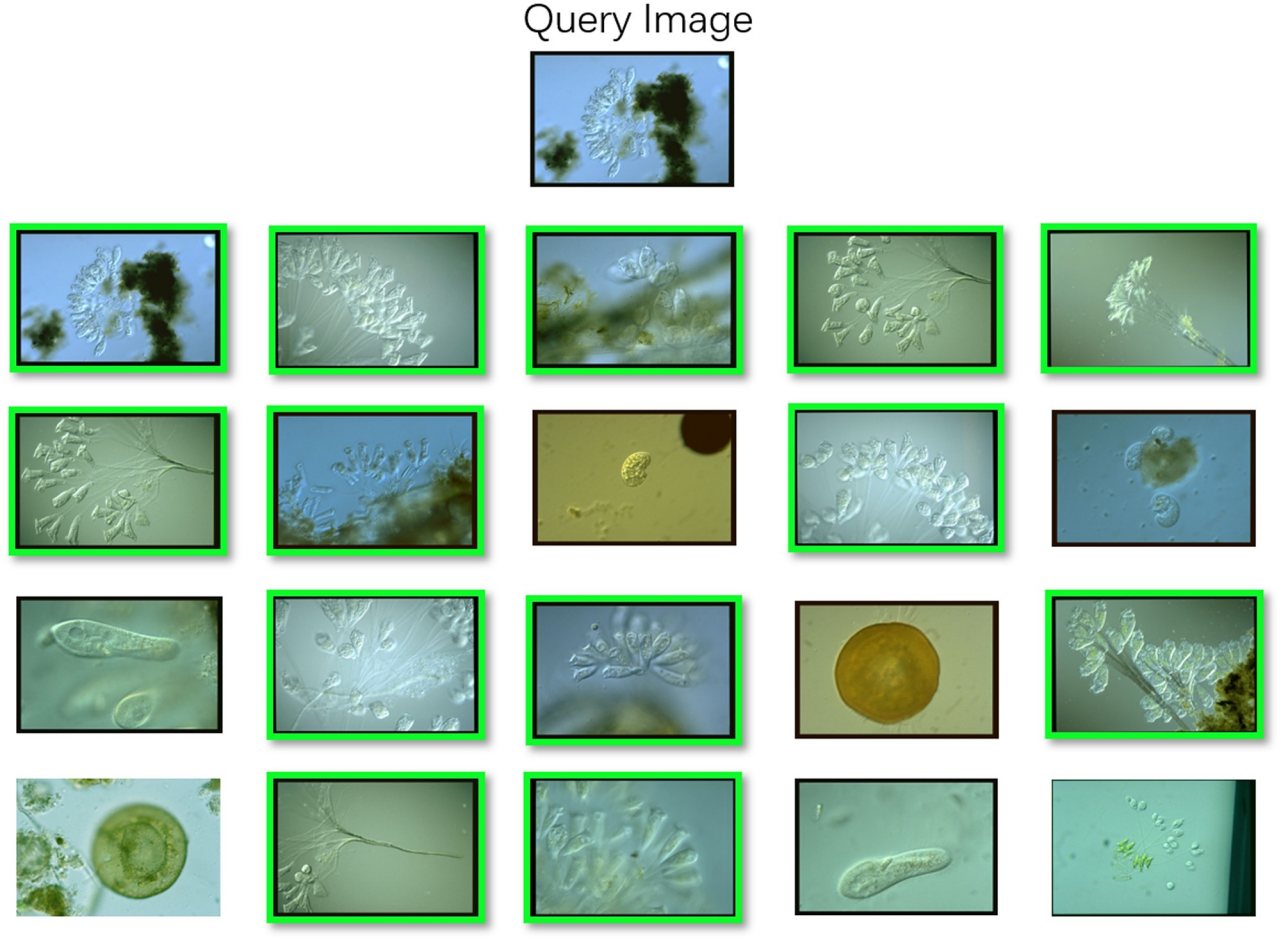
An example of image retrieval results with GLCM using EMDS-5.

We extract a total of four texture features, GLCM, GGCM, HOG and LBP to test the EMDS-5 image retrieval evaluation function. An example of the image retrieval results based on texture features is shown in Fig 9.

**Fig 10:**
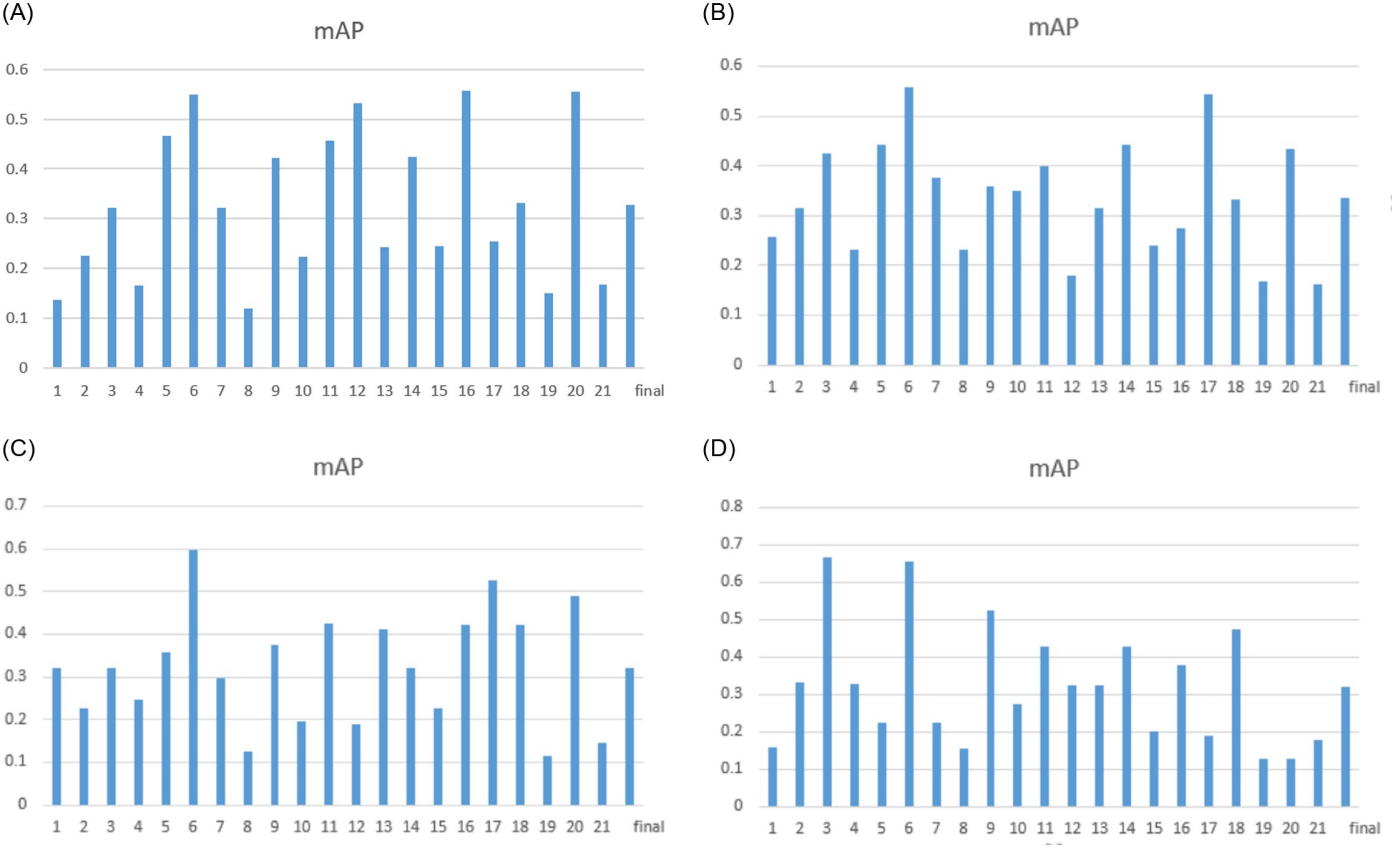
A comparison of image retrieval results with four texture features using EMDS-5.

Furthermore, the retrieval results of four texture features are demonstrated in Fig 10.

### 7.2 Deep learning feature based image retrieval using EMDS-5

**Fig 11:**
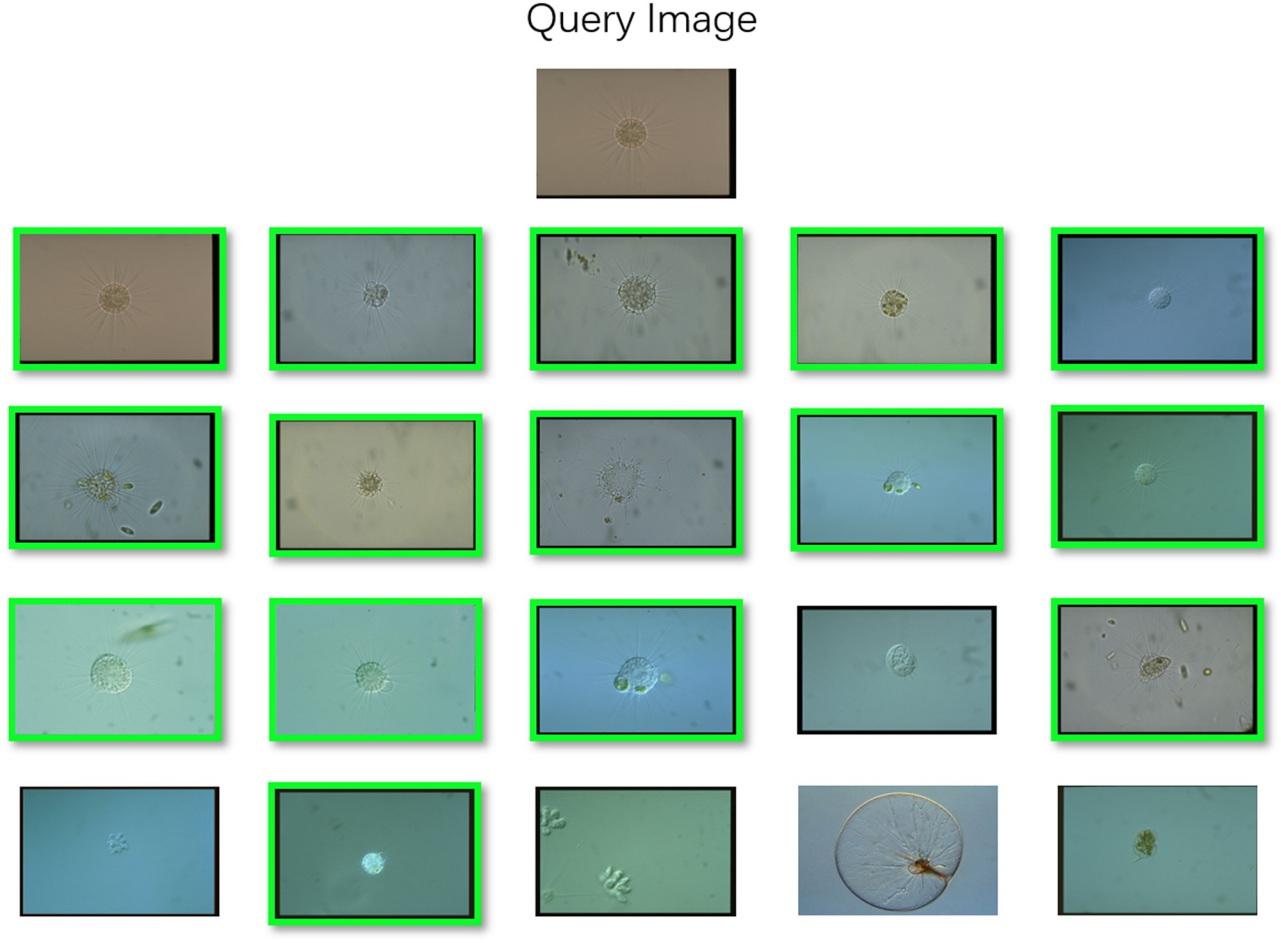
An example of image retrieval results based on VGG16 feature using EMDS-5.

We first extract VGG16 features and Resnet50 features. Then, the selected feature vectors are the feature vectors of the last layer of the respective network. The dimension is 1 × 1000. The following figure is an example of retrieval results based on deep learning features. An example of retrieval results based on deep learning features is shown in Fig 11.

**Fig 12:**
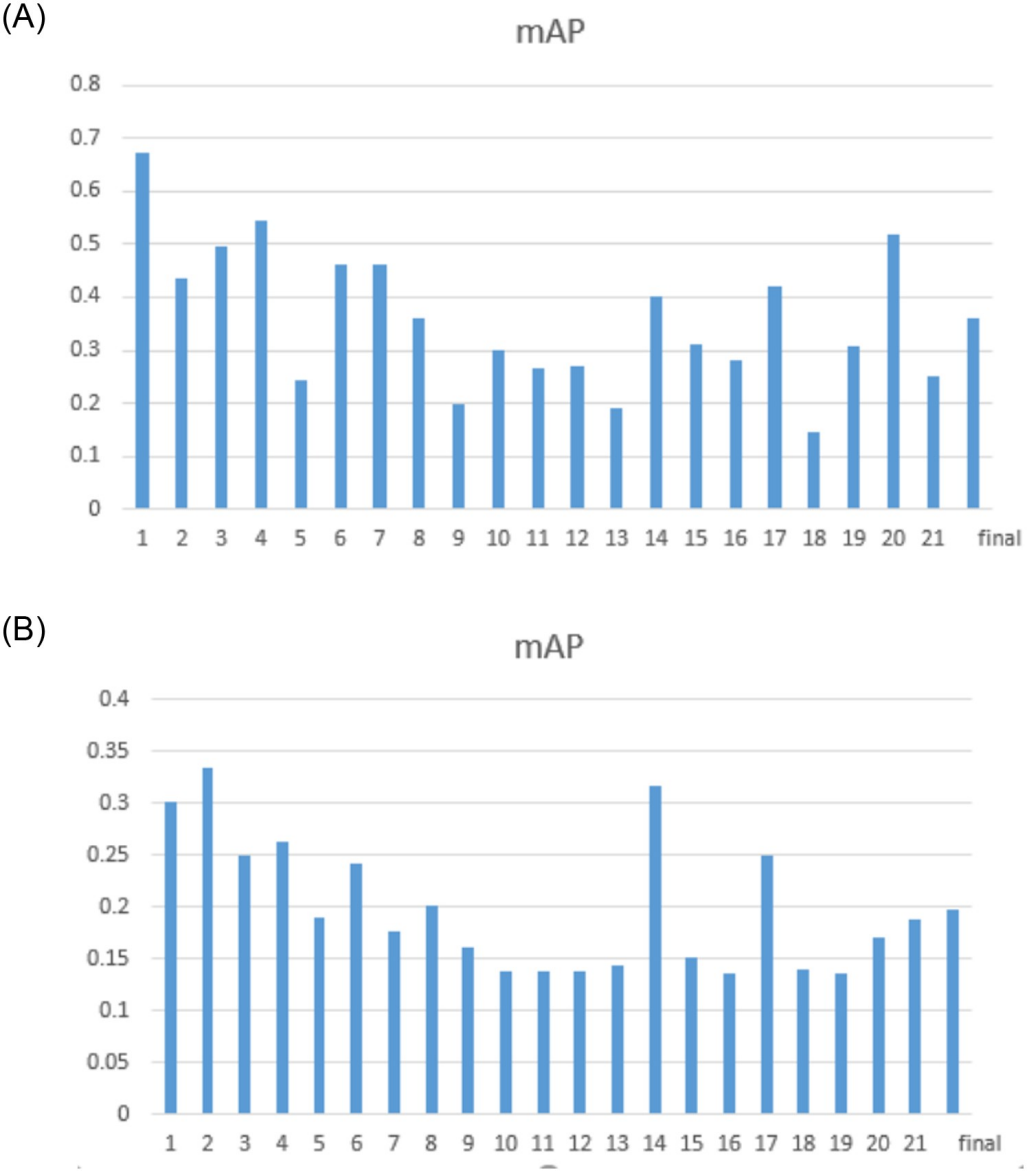
A comparison of image retrieval results with two deep learning features using EMDS-5.

Furthermore, the image retrieval results with two deep learning features are shown in Fig 12.

We calculate the variance of the mAP based on texture feature image retrieval and the variance of the mAP based on deep learning feature image retrieval. The result we get is that the variance of the image retrieval results based on deep learning features is smaller, which shows that the results of deep learning feature image retrieval are more stable. By comparing the results of different retrieval methods, we can know that EMDS-5 images can be effectively applied to various image retrieval tests and evaluations.

## 8 Conclusion and future work

EMDS-5 is a microscopic image dataset containing 21 classes of EMs. EMDS-5 contains original image and GT images of each EM. GT images include single-object GT images and multi-object GT images. Each original image has two corresponding GT images. Each microorganism class has 20 original images, 20 single-object GT images and 20 multi-object GT images. EMDS-5 has the function of testing the denoising effect. When testing the denoising effect of EMDS-5, we add 13 kinds of noise, such as Possion noise and Gaussian noise, and use nine kinds of filters to test the denoising effect of various noises. EMDS-5 can also evaluate the results of edge detection methods. We adopt six edge detection methods and use two evaluation indexes to evaluate the detection results and get good results. In terms of image segmentation, EMDS-5 can detect the results of image segmentation due to its single-object GT image and multi-object GT image. So we do the testing with two parts: single-object image segmentation and multi-object image segmentation. In the single-object image segmentation part, we use six methods such as GrubCut and MRF to segment the original images. In terms of multi-object image segmentation, we use *k*-means and U-Net methods for segmentation. We extract nine features from the images in EMDS-5, such as RGB, HSV, GLCM, HOG. We use the LIBSVM classifier to evaluate the results of the extracted features. In the test, we randomly select ten images of each type of EMs as the training set and ten images as the test set. In terms of classification, we use VGG16 features to test different classifiers such as LIBSVM, KNN, RF. In terms of image retrieval, we divide image retrieval based on texture features and image retrieval based on deep learning features. In terms of texture features, we select four features, GLCM, GGCM, HOG and LBP, to test separately. In the deep learning feature, we select two deep learning features, VGG16 feature and Resnet50, for retrieval. We select the last layer of features of these two deep learning networks as feature vectors. We use mAP as an evaluation index to detect the quality of retrieval.

In the future, we will expand the types of microorganisms and increase the number of images of each microorganism class. We hope that we can use the EMDS database to achieve more functions in the future.
